# The Effects of Transcranial Direct Current Stimulation in Obsessive–Compulsive Disorder Symptoms: A Meta-Analysis and Integrated Electric Fields Modeling Analysis

**DOI:** 10.3390/biomedicines11010080

**Published:** 2022-12-29

**Authors:** Bianca Silva Pinto, Beatriz Araújo Cavendish, Pedro Henrique Rodrigues da Silva, Paulo Jeng Chian Suen, Kalian Almeida Pereira Marinho, Leandro da Costa Lane Valiengo, Marie-Anne Vanderhasselt, André Russowsky Brunoni, Laís Boralli Razza

**Affiliations:** 1 Serviço Interdisciplinar de Neuromodulação (SIN), Instituto de Psiquiatria do Hospital das Clínicas HCFMUSP, Faculdade de Medicina, Universidade de São Paulo, São Paulo 05403-903, Brazil; 2 Programa de Fisiopatologia Experimental, Faculdade de Medicina, Universidade de São Paulo, São Paulo 05403-903, Brazil; 3 Laboratório de Neurociências (LIM-27), Instituto de Psiquiatria do Hospital das Clínicas HCFMUSP, Faculdade de Medicina, Universidade de São Paulo, São Paulo 05403-903, Brazil; 4 Department of Head and Skin, Psychiatry and Medical Psychology, Ghent University Hospital, Corneel Heymanslaan, 9000 Ghent, Belgium; 5 Ghent Experimental Psychiatry (GHEP) Lab, 9000 Ghent, Belgium; 6 Departamento de Clínica Médica, Faculdade de Medicina, Universidade de São Paulo, São Paulo 05403-903, Brazil

**Keywords:** randomized controlled trials, open-label trials, transcranial direct current stimulation, noninvasive brain stimulation, obsessive–compulsive disorder, computer modeling, electric field, regions of interest, meta-analysis, systematic-review

## Abstract

Transcranial direct current stimulation (tDCS) has been showing promising effects for the treatment of obsessive–compulsive disorder (OCD), but there is still no conclusion on its efficacy for this disorder. We performed a systematic review and meta-analysis of trials using tDCS for OCD and a computer modeling analysis to evaluate the electric field (EF) strengths of different electrode assemblies in brain regions of interest (ROIs) (PROSPERO-42021262465). PubMed/MEDLINE, Embase, Cochrane Library and Web of Science databases were searched from inception to 25 September 2022. Randomized controlled trials (RCTs) and open-label studies were included. The primary aim was the effect size (Hedges’ g) of continuous outcomes and potential moderators of response. For EF modeling, SimNIBS software was used. Four RCTs and four open-label trials were included (*n* = 241). Results revealed a large effect of tDCS in the endpoint, but no significant effect between active and sham protocols. No predictor of response was found. EF analysis revealed that montages using the main electrode over the (pre)supplementary motor area with an extracephalic reference electrode might lead to stronger EFs in the predefined ROIs. Our results revealed that tDCS might be a promising intervention to treat OCD; however, larger studies are warranted.

## 1. Background

Obsessive–compulsive disorder (OCD) is a disabling psychiatric condition, characterized by the presence of recurrent distressful images or intrusive/unwanted thoughts (obsessions) that are often associated with anxiety and/or compulsions performed in response to relieve the discomfort caused by the obsessions [1]. Worldwide, the lifetime prevalence of OCD in adults is 2–3% and is often underdiagnosed and undertreated despite its significant impairment in daily functioning [2]. OC symptoms are likely to be related to glutamatergic and dopaminergic overactivity in frontostriatal pathways and diminished serotonergic and GABAergic neurotransmission in frontolimbic systems [3]. Moreover, neuroimaging studies in patients with OCD show abnormal activation toward hypo or hyperactivity in brain regions including the (pre)supplementary motor area (SMA), insula, cingulate gyrus and the prefrontal cortex (PFC). It has been reported that these aberrant neural activations might be reverted after successful treatments [4]. First-line treatments for this condition include psychotherapy (cognitive behavioral therapy, CBT) and pharmacotherapies [5,6,7]. Nonetheless, a significant percentage of patients (up to 40%) do not respond to the first-line interventions [8].

Other forms of interventions, such as neuromodulation techniques, have been increasingly investigated as a treatment for OCD [9]. Considering that neuromodulation therapies apply electric currents in the brain able to modulate local and network-level activities [10], current studies suggest that forms of (non)invasive brain stimulation can ameliorate OCD symptoms [11]. Interventions that alter electrical brain activity include transcranial magnetic stimulation (TMS), transcranial alternating-current stimulation (tACS), electroconvulsive therapy (ECT), deep-brain stimulation (DBS) and focused ultrasound [9,12]. Of these, only repetitive transcranial magnetic stimulation (rTMS) and deep brain-stimulation (DBS) have shown high levels of evidence in treating OCD [13,14]. For instance, recent studies showed that both rTMS and DBS can modulate the abnormal cortical–limbic activities that are involved in OCD [15,16].

Another form of noninvasive brain stimulation (NIBS) that has been increasingly investigated as a treatment for OCD is transcranial direct current stimulation (tDCS). tDCS is an easy-to-apply, low-cost intervention, with excellent tolerability and safety profiles [17]. The technique uses a low-intensity current (1 to 4 mA) usually applied through two electrodes (anode and cathode) on the scalp. The current passes through the skin, the bone and the cephalorachidian fluid until reaching the brain, being able to modulate cortical excitability [18]. It has been suggested that tDCS targeting brain regions that present abnormal activation may lead to a decrease in OC symptoms. A few clinical trials were conducted to investigate its effects on OCD symptoms, and these have shown promising results [19,20,21,22,23,24,25,26]. In fact, based on these studies, recent guidelines recommended anodal stimulation over the presupplementary motor area (pre-SMA) as possibly effective (Level C) for the treatment of OCD symptoms [2].

However, clinical studies performed so far have adopted heterogeneous tDCS protocols, such as different numbers of sessions, various current intensities/densities and a high variability in electrode montages [19,20,21,22,23,24,25,26,27]. For instance, the largest randomized clinical trial (RCT) performed to investigate the efficacy of tDCS for OCD targeted different brain areas and was not able to find any significant improvement in the acute treatment phase between sham and active tDCS protocols [22,24]. Therefore, there is still no conclusions concerning the efficacy of tDCS for OCD symptoms, and the optimal electrode montage for this treatment is still unknown. Moreover, no meta-analysis has been conducted to quantitatively investigate the overall efficacy of tDCS for OCD so far.

Therefore, due to the aforementioned gaps in the literature, we aimed to perform a systematic review and meta-analyses of clinical trials evaluating the efficacy of tDCS for the improvement of OCD symptoms and its potential moderators of response. The second aim was to conduct an EF modeling analysis to evaluate the EF strengths of different electrode montages in predefined regions of interest (ROIs), aiming to investigate whether there is an optimal tDCS montage for OCD treatment. Our study is important for the literature, as it provides a better understanding of the role of tDCS treatment for OCD and might provide important insights for the design of future clinical trials.

## 2. Materials and Methods

A systematic review was conducted from the first date available until 25 September 2022, on the PubMed/MEDLINE, Embase, Cochrane Library and Web of Science databases, with no language restriction. The search was conducted using the main terms: ‘obsessive compulsive disorder’, ‘OCD’, ‘transcranial direct current stimulation’, ‘tDCS’ and ‘clinical trial’ (Supplementary File S1). Experts in the field and tDCS trials included in a previous published systematic review [28] were also contacted. The study was preregistered in the International Prospective Register of Systematic Reviews (PROSPERO—reference CRD 42021262465—https://www.crd.york.ac.uk/PROSPERO/display_record.php? (accessed on 21 October 2021)”. Two authors (B.S.P. and L.B.R.) independently searched the studies and screened the titles and abstracts to identify the eligible trials by using Rayyan software [29]. Any disagreements were discussed with the coauthors and resolved through consensus.

### 2.1. Eligibility Criteria

In this systematic review and meta-analysis, we included only clinical trials investigating the effects of tDCS for the symptoms of OCD in adult patients. We included both open-label and RCTs that performed at least 10 active tDCS sessions and that enrolled participants with a diagnosis of OCD based on *the Diagnostic and Statistical Manual of Mental Disorders* (DSM) or the *International Statistical Classification of Diseases and Related Health Problems* (ICD) criteria. Studies had to have included at least 5 participants per arm and reported the results through the Yale-Brown Obsessive-Compulsive Scale (Y-BOCS). In addition, we included studies in which tDCS was applied as monotherapy or in association with other therapeutic interventions (e.g., psychotherapy or medication). Exclusion criteria were as follows: (a) case reports and case series studies; (b) studies presenting duplicated data; (c) other transcranial electric stimulation interventions than tDCS.

### 2.2. Quality Assessment

The methodological quality of the included studies was assessed by The Cochrane Risk of Bias Tool (RoB2) and by the Risk of Bias in Non-randomized Studies- of Interventions tool (ROBINS-I), recommended by the Cochrane Group to evaluate risk of bias in such studies. Two authors (B.S.P. and B.A.C.) independently assessed the risk of bias of each study [19,20,21,22,23,24,25,26,27,30,31]. The domains assessed in the RoB2 tool were selection bias, performance bias, attrition bias, detection bias and reporting bias according to a standardized criterion (Table S1). Meanwhile, the ROBINS-I assessed the domains related to baseline confounding factors, participant selection, misclassification of interventions, deviations from intended interventions, missing data, errors in the measurement of outcomes, selective reporting of results, confusion bias, selection bias, bias due to lack of data, bias in outcome measurement and bias in the selection of the reported result (Table S2). According to the RoB2 classification, risks of bias were categorized as ‘low’, ‘high’ and ‘some concerns’. The ROBINS-I tool classifies risk of bias as ‘low’, ‘moderate’, ‘serious’ or ‘critical’.

### 2.3. Data Extraction

Data extraction was performed by two authors (B.S.P. and B.A.C.) and checked by a third author (L.B.R.). Variables extracted were the following: (a) metadata: first author and year of publication; (b) information on outcomes: sample size, number of dropouts, mean and standard deviations (SDs) of Y-BOCS scale from the baseline and primary outcome of each study of both active and sham groups, when applicable; (c) tDCS parameters: number of sessions, session duration, current intensity, electrode position, study length and treatment strategy; (d) demographic data: mean age and gender.

If studies did not report any essential information, such as mean score, SDs or sample size, the corresponding author was contacted by email. If no reply was obtained, data were extracted from the article’s graphs with the WebPlotDigitizer [32], as recommended elsewhere [33]. Moreover, for within-subject studies, such as crossover trials, we considered only data from the first phase to avoid carry-over effects.

### 2.4. Outcomes

The first outcome of this study was to evaluate the clinical effects of active tDCS for OCD patients using the Y-BOCS scale. We used sample sizes, means and SDs of the OCD score scale of all studies and compared results from the baseline with endpoint scores. Secondly, we performed an analysis including only RCTs. For this analysis we used only endpoint data (means, SDs and sample sizes) from both sham and active groups. Potential response moderators via meta-regressions were investigated as secondary analysis.

The second outcome was to investigate the intensity of the EF in key brain regions associated with OCD symptoms using the tDCS electrode montages applied in each included study. The methods used to perform the EF modeling analysis can be found below.

### 2.5. Electric Field Modeling

Electric field modeling was performed using SimNIBS (v3.1, Danish Research Centre for Magnetic Resonance, Copenhagen, Denmark) [34], a piece of free and open-source software that allows simulation of tDCS-induced EF in the individual brain. This method allows for an approximation of the ‘real’ current distribution in the brain and has been shown to accurately predict real field magnitudes [35]. First, in order to estimate the EF values in the brain, a high-resolution head model of a subject in which the modeling will be performed is necessary. This head model is created based on a T1-weighted MRI anatomical image of the subject by using the *headreco* pipeline in SimNIBS [36]. This pipeline is dependent on MATLAB software (version R2021 was used), and it was chosen since it is the most recent tool, with a segmentation that includes the neck for positioning extracephalic electrodes. It segments five tissue types based on the provided structural MRI scan: white matter, gray matter, cerebrospinal fluid (CSF), bone and scalp. Then, it creates a 3D tetrahedral mesh structure of each segmented tissue, which allows for the simulation of the EF.

We manually verified the segmentation to check for possible errors in the established boundaries between tissues. Conductivity values used for each tissue type were as follows: 0.126 S/m for white matter, 0.275 S/m for gray matter, 1.654 S/m for CSF, 0.01 S/m for bone and 0.465 S/m for scalp. The resulting 3D head model was then used to simulate the EF distribution resulting from the various tDCS montages used in each study protocol included in this analysis. This was done by placing simulated electrodes on the head model and setting the simulated electric current intensity according to the stimulation protocol. We followed the information reported in each study included in our systematic review. In the cases where the direction of the rectangular electrodes were not specified (e.g., toward Cz or not), corresponding authors were contacted by email. When no response was obtained, the EF modeling was performed using two different electrodes montages (i.e., toward Cz and horizontally) to investigate the EF strength between them. The montages presenting the strongest EF were included in our analysis (Table S3). For all simulations, we used a head model of an OCD patient obtained from the database of the Magnetic Resonance Imaging (MRI) laboratory from the Institute of Psychiatry, University of São Paulo, Brazil. This participant gave written consent for data collection and publication. The MRI imaging was acquired on a 3T Achieva MRI scanner (Philips Healthcare, Best, Netherlands) with the following parameters: repetition time = 10, 200 ms, echo time = 103 ms, flip angle = 90°, field of view = 256 × 256 × 140 mm^3^, matrix size = 128 × 128, 70 slices, 2.0 mm slice thickness, 64 gradient directions with a b-value of 1000 and one b = 0 volume.

We investigated the EF strength in predefined bilateral ROIs, which were as follows: SMA, pre-SMA, dorsolateral prefrontal cortex (DLPFC), insula and anterior cingulate cortex (ACC). To define these regions, we used a comprehensive multimodal whole-brain parcellation atlas [37], and the calculated EF was the mean of the subregions included in each ROI (Table S4). All ROIs used in our study involved brain regions implicated in OCD, according to van Den Heuvel [38].

### 2.6. Data Analysis

Quantitative analyses were performed through Stata 17 software (StataCorp LLC) by using the *meta* command. Univariate meta-regressions were performed using the *metareg* command. Considering the high heterogeneity among studies, a random-effects model (restricted maximum-likelihood method) instead of fixed-effects was used [39]. High heterogeneity was considered when the *I*-squared *(I*^2^*)* was >50%, as proposed elsewhere [40]. Moreover, given that the studies had small sample sizes, the Hedges’ g was the effect size measured. Effects sizes of up to 0.35, between 0.35 to 0.7 and >0.70 were considered, respectively, small, moderate and large. Funnel plot and the Egger regression intercept test were applied to investigate small-study effects.

Linear regression models (‘stats’ package) using R software (version 4.2.0) were performed to investigate EF strength based on the predefined ROIs. ‘Montage’ was considered the dependent variable, while ‘ROI’ was the independent variable. As five ROIs were investigated bilaterally, ten linear regressions models were performed. Moreover, based on a previous study [41], D’Urso et al., 2016 (cathode) [23] was used as the reference montage. Therefore, the EF strength of each ROI was found to be significant when differing from D’Urso et al.’s 2016 (cathode) values. The adopted significance threshold was an alpha level of 0.05 [20,21].

## 3. Results

**Figure 1:**
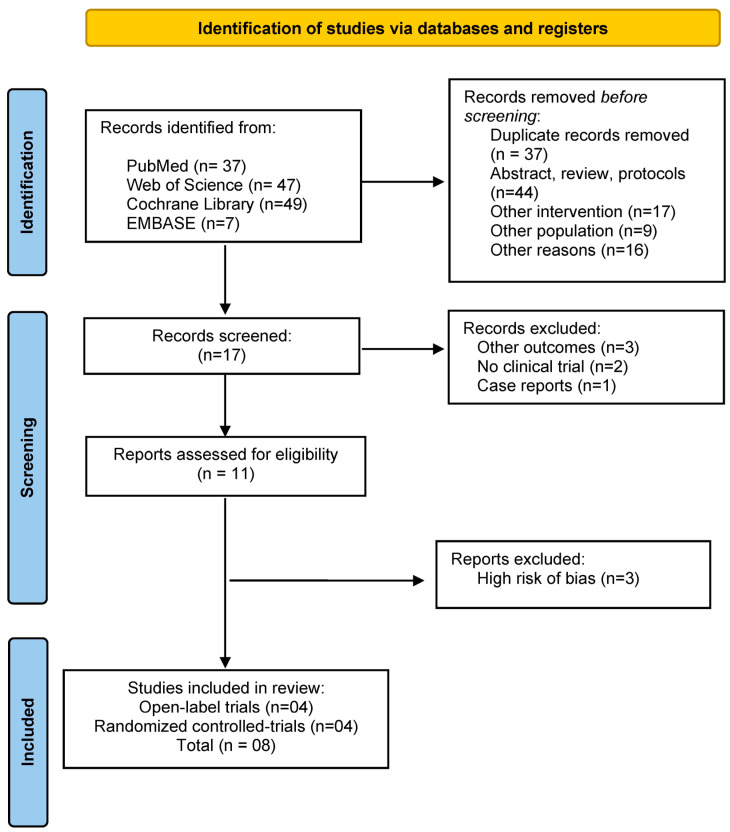
Flowchart of the study selection process.

Our search initially yielded a total of 140 references, of which 132 were excluded for several reasons [Figure 1] and 11 met inclusion criteria [19,20,21,22,23,24,25,26,27,30,31]. Three studies were excluded from our analysis because they presented a high risk of bias, mainly involving methodological issues [27,30,31] (Tables S1 and S2). Therefore, eight studies (9 datasets) were included in our meta-analysis. Among them, there were 4 open-label trials and 4 RCTs with an overall 241 participants (mean age: 36.14; SD: 11.07; female: 53.9%) (Table 1). A total of 165 participants received active treatment, and 76 received sham intervention. Quality assessment revealed that 75%, 25% and 0% of the included RCTs presented low, some concerns and high risk of bias, respectively, while all open-label studies presented a moderate risk of bias (Tables S1 and S2).

The included studies are described in detail as follows: 

Bation et al., 2016 conducted an open-label study that included 8 patients with treatment-resistant OCD, who received 10 sessions (twice a day) using a 2 mA current for 20 min. The cathode was placed over the left OFC and anode over the right cerebellum. There was a decrease of 26.4% in the Y-BOCS score after active tDCS which lasted through the 3-month follow-up [21].

D’urso et al., 2016 conducted an open-label cross-over study applying cathodal and/or anodal tDCS over the pre-SMA with an extracephalic reference electrode. Twelve patients were included and received 10 sessions of anodal (*n* = 6) or cathodal (*n* = 6) stimulation of 2 mA for 20 min. At the end of the study, there was a statistically significant decrease in the mean Y-BOCS scores of patients undergoing cathodal tDCS but not of those undergoing anodal tDCS [23].

In the open-label study conducted by Kumar et al., 2019, authors aimed to investigate the response of tDCS as an adjuvant treatment of pharmacotherapy in patients with treatment-resistant OCD. Twenty patients with treatment-resistant OCD were included. Participants received 20 sessions of tDCS with cathode over the SMA and anode over the right occipital area. An improvement of >35% change in Y-BOCS score was observed in 15% of participants between groups. Authors concluded that cathodal tDCS over SMA may be a useful approach to treatment-resistant OCD patients [26].

In the study by Germeneau et al. (2020), an open-label study was conducted aiming to assess the therapeutic efficacy and tolerability of cathodal tDCS on the SMA and anode over the right occipital area in treatment-resistant OCD patients. Twenty patients received 10 sessions of tDCS of 2 mA for 30 min. The study findings revealed a significant decrease in YBOCS scores between baseline and endpoint, with 6 responders. At one and thirty months of follow-up, five (24%) and 3 patients (15%) were, respectively, considered responders [19].

In the RCT conducted by Bation et al., 2019, 21 patients with OCD received ten tDCS sessions (2 daily sessions) of 2 mA/20 min. Findings revealed that the sham tDCS was superior to active tDCS in decreasing the OCD symptoms after ten sessions of tDCS. However, no significant differences were observed between the active and sham groups in terms of changes in the clinical scale score or in the number of responders during the follow-up phase [20].

In the RCT by Gowda et al., 2019, they investigated the efficacy of tDCS in reducing symptoms in OCD patients resistant to serotonin reuptake inhibitor treatment by applying anodal tDCS over the pre-SMA and cathodes over the right supraorbital area. Twenty-five patients received 10 tDCS sessions (2 daily sessions) of 2 mA for 20 min. Findings revealed that response rate was significantly higher for the active tDCS (4 out of 12) compared to the sham tDCS (0 out of 13) [25].

Silva et al., 2021 conducted an RCT in which they investigated the effectiveness of tDCS as an add-on treatment for 43 treatment-resistant OCD patients that received 20 consecutive tDCS sessions of 2 mA for 30 min with the cathode applied over the SMA and the anode over the left deltoid. Results showed that active tDCS was superior to sham in reducing OCD symptoms (Cohen’s d: 0.62; 06–1.18), *p* = 0.03) [22].

In the Yoosefee et al., 2020 RCT, they aimed to assess the safety and efficacy of tDCS as adjunctive therapy with fluoxetine in 60 subjects diagnosed with moderate to severe OCD. Two groups were examined: a tDCS plus fluoxetine (experimental arm) group and a fluoxetine alone (sham control arm) group. The anode was placed over the left DLPFC (Fp3) while the cathode was placed over the right orbitofrontal cortex (F8). tDCS was administered for 20 min/2 mA three times a week for 8 weeks. No difference in OCD symptoms between the experimental and control group was found [24].

### 3.1. Primary Outcome

**Figure 2:**
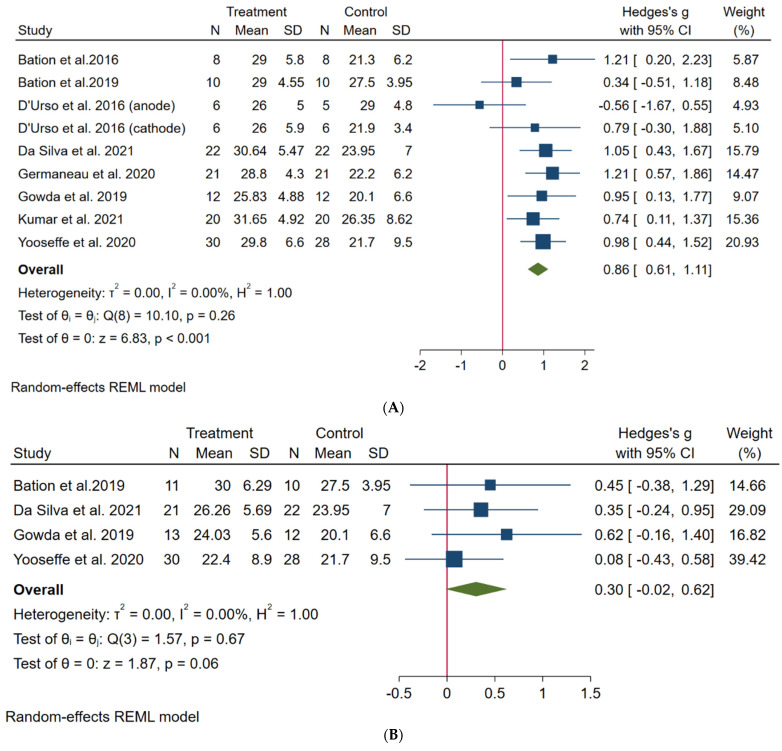
Forest plots. (**A**) Primary outcome: comparison between baseline and endpoint of active groups [19,20,21,22,23,24,25,26]. (**B**) Secondary outcome: comparison between endpoints of both active and placebo groups [20,22,24,25].

We calculated the effect sizes between baseline and endpoint of Y-BOCS scores for each study. The analysis showed a large effect size favoring the end of treatment (k = 8, Hedges’s g = 0.86, 95% confidence interval (CI): 0.61; 1.11). Low heterogeneity was observed (*I*^2^ = 0.00%) [Figure 2A]. The funnel plot showed deviation of one small study toward a negative effect [Figure S1]; however, the Egger test revealed no evidence of publication bias (t = −1.46, *p* = 0.14).

Secondary analysis considering only the RCTs revealed that active tDCS was not superior to sham at the endpoint (k = 4, *g* = 0.30, 95% CI: −0.02; 0.62, *I*^2^ = 0.00) [Figure 2B]. Comparisons between the baseline and endpoint of both active and sham groups showed that both protocols presented improvements at the endpoint, with a larger effect size for the active group [Figure S2].

### 3.2. Meta-Regression Analysis

Meta-regression analyses revealed no variables associated with the primary outcome (Table 2).

### 3.3. Electric Field Modeling Analysis

**Figure 3:**
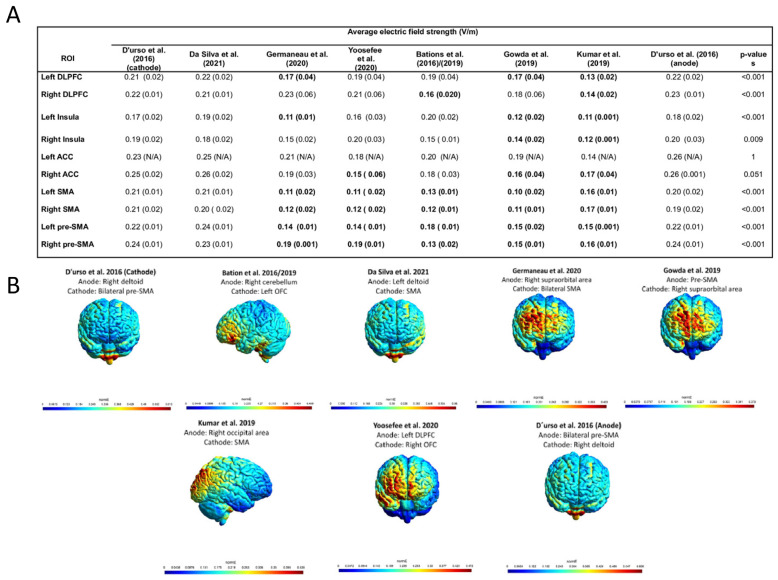
Electric field modeling analysis [19,20,21,22,23,24,25,26]. (**A**) Mean and standard deviation of electric field strength of montages across different brain regions of interest. (**B**) Electric field distribution based on the different montages used. Note: For this analysis, we used the D’Urso et al. study (cathode) as reference, as this montage has already been found to produce stronger EFs in regions of interest for OCD [41,42]. A higher mean represents a stronger EF in the regions of interest. Results displayed in bold font represent significance in comparison to D’Urso et al., 2016 (cathode). Therefore, Da Silva et al., 2021 [22] and D’Urso et al., 2006 (anode) [23] did not show any EF strength differences in comparison to the reference montage. Abbreviation: ACC—anterior cingulate cortex; N/A—not applicable (For the left ACC analysis, the electric field modeling was able to quantify only one subregion. Therefore, no standard deviation is presented.); ROI—region of interest; SMA—supplementary motor area. *p*-values represent the linear regression results. *p*-values ≤ 0.05 were considered significant.

According to the EF modeling analysis, montages applying the main electrode over the (pre)-SMA with an extracephalic reference electrode were able to induce overall stronger EFs on the evaluated ROIs in comparison to other montages [Figure 3A,B and Supplementary File S7].

## 4. Discussion

To our best knowledge, this is the first meta-analysis investigating the acute effects of tDCS for the treatment of patients with OCD and its potential moderators. Eight studies were included (4 open-label and 4 randomized controlled trials, *n* = 241) presenting no serious overall risk of bias. No evidence of publication bias was found. Main result showed a large effect for tDCS when comparing measures from baseline to endpoint, but no difference was found when active tDCS was compared to sham. Moreover, meta-regression revealed no variables significantly influencing the effect of tDCS. EF modeling analyses showed that montages using the main electrode targeting the (pre)-SMA with a reference extracephalic electrode can generate greater EF intensity in specific brain structures related to OCD in comparison to other montages.

The results of this meta-analysis show that tDCS can be a promising intervention to reduce OCD symptoms. However, these effects should be interpreted with caution since active tDCS compared to sham did not show a significant effect. A few reasons might explain the lack of positive effect when comparing active to sham tDCS. First, there were only four randomized controlled trials included in our meta-analysis that were limited by overall small sample sizes. Second, there was a high heterogeneity of tDCS protocols across these four studies, especially in terms of electrode montage. For instance, none of the studies applied the cathode over the same targeted brain area. Therefore, it seems that both small the sample sizes and high variability of protocols are significant limitations that restrict our conclusions regarding the effects of tDCS as a treatment of OCD.

As the high variability of tDCS montages is still a limitation for OCD treatment, our results revealed that montages using the main electrode over the (pre)-SMA with an extracephalic reference electrode can induce an overall stronger EF in the regions of interest in comparison to the other montages. This finding is similar to a previous study that performed EF modeling analysis using five different tDCS montages for OCD patients and showed that targeting the SMA with the cathode could deliver stronger electric fields to some cerebral structures of interest, such as the inferior ventral striatum, medial prefrontal cortex, the dorsolateral prefrontal cortex and the basal ganglia [23]. However, in our review three trials targeted the (pre)-SMA using distinctives polarities (i.e., an anode over the SMA with an extracephalic cathode and a cathode over the SMA with an extracephalic anode). As we did not assess the direction of the EF, we are not able to confirm whether an anode or cathode over the (pre)-SMA is the best option for improving OCD symptoms. However, it is important to underscore that all studies used a current intensity of 2 mA, as well as session duration of 20 or 30 min. This is important for replicability of future clinical trials. Furthermore, as no response prediction was found through meta-regression analysis with clinical and demographic characteristics, the EFs results may help to elucidate how to increase the effects of tDCS for OCD and also reduce the variability between studies.

Another reason to target the SMA is its relation with the sensorimotor circuit—a circuitry that involves cortical (i.e., SMA) and subcortical (putamen, thalamus and insula) areas associated in the generation and control of motor behaviors and integration of sensory information [38]. Atypicalities related to this circuitry may cause patients to experience sensory phenomena, a common symptom that generates aversive or uncomfortable sensations or perceptions that drive repetitive behaviors and is reported by approximately 70% of patients [38].

Finally, besides tDCS, another NIBS approach is rTMS, which uses a focused EF to change brain activity [43]. While tDCS research is still in its infancy as an OCD treatment, rTMS treatment has already been approved by the US Food and Drug Administration (FDA) using different types of coils, such as double cone and deep TMS [44,45]. In fact, a recent meta-analysis investigating the efficacy of NIBS for mental disorders [46] shows a discrepancy between tDCS and rTMS trials for OCD, as there were twenty-six studies included using rTMS and only two for tDCS after the inclusion criteria were applied [46]. Although the evidence suggests that rTMS can indeed ameliorate OCD symptoms while tDCS effects are still unclear, more clinical studies using tDCS must be performed. Moreover, tDCS presents several advantages compared with rTMS, such as lower costs, self-administration and the possibility of being applied at home. Home-based applications are particularly appealing for recruiting more participants more quickly and for clinical study [47,48,49,50] or when using tDCS for longer follow-up periods. In fact, studies evaluating the follow-up effects of tDCS for OCD observed that the effects can persist for a long period or even increase after an acute tDCS treatment [22]; in this case, maintenance tDCS sessions would be advised. However, to our best knowledge there is still no study evaluating the maintenance effects of tDCS for OCD. For the maintenance phase, tDCS also presents an advantage over rTMS since home use devices can avoid the daily displacement to specialized centers, travel costs and disruptions to daily activities [50].

## 5. Limitations and Future Directions

This study has some limitations that should be underscored. First, the sample size of all included studies is small, and the tDCS protocols used were heterogeneous, which might have biased our results. Moreover, three RCTs that met inclusion criteria were excluded due to a high risk of bias. Taken together, these points highlight the urgent need for larger, high-quality methodological trials of tDCS for OCD. Second, the open-label studies included in the meta-analysis also could have biased the overall effect size regarding effects of endpoint in comparison to baseline scores since participants were not blinded, which could have led to an enhancement of the nonspecific effects of the treatment. Third, we performed only an aggregate data meta-analysis, which has poorer performance than does an individual patient data meta-analysis, especially for identifying moderators of the outcome [51]. Fourth, EF modeling analysis was performed using only one head, which is limited in accounting for interindividual anatomical variability. Fifth, it should be noted that two studies included in this review did not report the electrode direction [30,31] and our analysis was performed based on the electrodes’ direction that presented stronger EFs in the ROIs. Therefore, as different electrode directions can cause different EF strengths, future studies should account for this variability. Finally, we did not assess the long-term effects of tDCS for OCD. To the best of our knowledge, there are only four studies investigating the prolonged effects of tDCS. and they used different study designs for the acute phase (e.g., RCT and open label) which makes the analysis very heterogeneous and difficult to interpret. Therefore, we decided not to perform analysis for the follow-up data.

For future perspectives, we believe that the findings of this meta-analysis and electric field modeling analysis can encourage future large trials using tDCS for OCD. First, to the best of our knowledge this is the first time that the effects of tDCS have been quantitatively evaluated for OCD in an acute treatment phase; therefore, the results presented here reinforce previous literature suggesting that tDCS can be beneficial for OCD symptoms. Second, our study provides evidence that targeting the (pre)-SMA might deliver stronger electric fields in circuits involved in OCD disorders, which should be replicated in future studies and might reduce heterogeneity in tDCS clinical parameters for OCD. Third, the results of the electric field modeling analysis are not only important from a clinical point of view, but also a translational one since future experimental/neuroimaging studies using tDCS can be based on the results of this work and focus on the SMA as a brain target area to modulate dysfunctional brain networks related to OCD. Fourth, no study included in this meta-analysis investigated the benefits of OCD symptom provocation before a tDCS session. As exposure therapies before rTMS intervention have been yielding positive results [52], future studies are advised to investigate its benefits in combination with tDCS. Fifth, longer follow-up trials are encouraged in order to clarify the maintenance effects of tDCS for OCD.

## 6. Conclusions

To conclude, this systematic review and meta-analysis retrieved eight clinical studies (4 open-label trials and 4 randomized trials with an overall 241 participants) investigating tDCS as a treatment for OCD symptoms. The findings show that a course of active tDCS can be beneficial for OCD patients; however, no differences were found in comparison to sham. Although studies presented a high variability in tDCS montages, the EF modeling results suggest that montages using the main electrode over the (pre)-SMA with an extracephalic reference electrode can deliver stronger EFs to regions associated with OCD symptoms. Finally, although the findings of this meta-analysis show that active tDCS might improve OCD clinical gains, clinical trials using less heterogeneous tDCS parameters are still needed. Therefore, our findings encourage future large trials to focus on the (pre)-SMA as a brain target area for tDCS in OCD treatments and to apply tDCS for long follow-up periods to further elucidate the effectiveness of tDCS for OCD patients.

## Figures and Tables

**Table 1:** Summary of the included studies.

Demographic Data				Clinical Data		tDCS Strategy						
Reference	Sample Size	Mean Age	% Female	Treat. Strategy	TRD	Number of Sessions	Current Intensity	Duration (min)	Electrode Position	Electrode Size (cm^2^)	Study Design	Risk of Bias
Bation et al. (2016) [21]	8	44.2 (13.8)	75	Add-on	Yes	10	2	20	Anode: right cerebellum Cathode: left OFC	35	Open label trial	Moderate
Bation et al. (2019) [20]	21	43 (15.9)	57.14	Add-on	Yes	10	2	20	Anode: right cerebellum Cathode: left OFC	35	RCT	Low
Da Silva et al. (2021) [22]	44	37.65 (11.59)	60.46	Add-on	Yes	20	2	30	Anode: left deltoid Cathode: SMA	25	RCT	Low
D’Urso et al. (2016) [23] (cathode)	6	N/I	N/I	Add-on	Yes	10	2	20	Anode: right deltoid Cathode: bilateral pre-SMA	35 and 25	Partial cross-over, repeated measures	Moderate
D’Urso et al. (2016) [23] (anode)	6	N/I	N/I	Add-on	Yes	10	2	20	Anode: bilateral pre-SMA Cathode: right deltoid	25 and 35	Partial cross-over, repeated measures	Moderate
Germaneau et al. (2020) [19]	21	42.7 (13)	38.1	Add-on	Yes	10	2	30	Anode: right supraorbital area Cathode: bilateral SMA	35	Open label trial	Moderate
Gowda et al. (2019) [25]	25	28.37 (5.51)	16	Add-on	Yes	10	2	20	Anode: pre-SMA Cathode: right supraorbital area	35	RCT	Some Concers
Kumar et al. (2021) [26]	20	21.6 (7.64)	45	Add-on	Yes	20	2	20	Anode: right occipital area Cathode: SMA	25*	Open label trial	Moderate
Yoosefee et al. (2020) [24]	60	37.25 (24.1)	81.6	Augmentation	N/I	24	2	20	Anode: left DLPFC Cathode: right OFC	35	RCT	Low

Abbreviations: N/I, not informed; RCT, randomized controlled trials; TRD, treatment-resistant OCD; SMA, supplementary motor area; OFC, orbitofrontal cortex; DLPFC, dorsolateral prefrontal cortex. Note: Eight studies were included in this systematic review and meta-analysis, of which four are randomized controlled trials and four are open-label trials.

**Table 2:** Results of univariate meta-regression analyses.

	Coef (B)	95% CI		*p*
		Lower Limit	Upper Limit	
Augmentation Strategy	−0.02	−0.88	0.46	0.54
Sessions per day	0.49	−0.18	1.18	0.15
Session duration	−0.27	−0.09	0.41	0.43
Number of weeks	−0.05	−0.16	0.48	0.27
Session total	−0.03	−0.08	0.01	0.18

Note: Meta-regressions were performed using one variable per time due to the relatively low sample size included in this meta-analysis. Statistical significance was indicated by *p* ≤ 0.05. Therefore, no variable was found to be statistically significant. Augmentation strategy: tDCS combined with concurrently commenced other interventions (i.e., pharmacotherapy). Abbreviation: CI—confidence interval.

## Data Availability

Not applicable.

## Supplementary Materials

The following supporting information can be downloaded at: https://www.mdpi.com/article/10.3390/biomedicines11010080/s1. References [19,25,30,31,37] are cited in the supplementary materials.

## References

[1] Brock H., Hany M. (2021). Obsessive-compulsive disorder. StatPearls.

[2] Stein D.J., Costa D.L.C., Lochner C., Miguel E.C., Reddy Y.C.J., Shavitt R.G., van den Heuvel O.A., Simpson H.B. (2019). Obsessive-Compulsive Disorder. Nat. Rev. Dis. Prim..

[3] Tanaka M., Szabó Á., Spekker E., Polyák H., Tóth F., Vécsei L. (2022). Mitochondrial Impairment: A Common Motif in Neuropsychiatric Presentation? The Link to the Tryptophan-Kynurenine Metabolic System. Cells.

[4] Battaglia S., Harrison B.J., Fullana M.A. (2022). Does the Human Ventromedial Prefrontal Cortex Support Fear Learning, Fear Extinction or Both? A Commentary on Subregional Contributions. Mol. Psychiatry.

[5] Hirschtritt M.E., Bloch M.H., Mathews C.A. (2017). Obsessive-Compulsive Disorder: Advances in Diagnosis and Treatment. JAMA.

[6] Denys D. (2006). Pharmacotherapy of Obsessive-Compulsive Disorder and Obsessive-Compulsive Spectrum Disorders. Psychiatr. Clin. N. Am..

[7] Denys D., Mantione M., Figee M., van den Munckhof P., Koerselman F., Westenberg H., Bosch A., Schuurman R. (2010). Deep Brain Stimulation of the Nucleus Accumbens for Treatment-Refractory Obsessive-Compulsive Disorder. Arch. Gen. Psychiatry.

[8] Pittenger C., Bloch M.H. (2014). Pharmacological Treatment of Obsessive-Compulsive Disorder. Psychiatr. Clin. N. Am..

[9] Kammen A., Cavaleri J., Lam J., Frank A.C., Mason X., Choi W., Penn M., Brasfield K., Van Noppen B., Murray S.B. (2022). Neuromodulation of OCD: A Review of Invasive and Non-Invasive Methods. Front. Neurol..

[10] Borgomaneri S., Battaglia S., Sciamanna G., Tortora F., Laricchiuta D. (2021). Memories Are Not Written in Stone: Re-Writing Fear Memories by Means of Non-Invasive Brain Stimulation and Optogenetic Manipulations. Neurosci. Biobehav. Rev..

[11] Begemann M.J., Brand B.A., Ćurčić-Blake B., Aleman A., Sommer I.E. (2020). Efficacy of Non-Invasive Brain Stimulation on Cognitive Functioning in Brain Disorders: A Meta-Analysis. Psychol. Med..

[12] Klimke A., Nitsche M.A., Maurer K., Voss U. (2016). Case Report: Successful Treatment of Therapy-Resistant OCD With Application of Transcranial Alternating Current Stimulation (tACS). Brain Stimul..

[13] Bergfeld I.O., Dijkstra E., Graat I., de Koning P., van den Boom B.J.G., Arbab T., Vulink N., Denys D., Willuhn I., Mocking R.J.T. (2021). Invasive and Non-Invasive Neurostimulation for OCD. Curr. Top. Behav. Neurosci..

[14] Trevizol A.P., Shiozawa P., Cook I.A., Sato I.A., Kaku C.B., Guimarães F.B., Sachdev P., Sarkhel S., Cordeiro Q. (2016). Transcranial Magnetic Stimulation for Obsessive-Compulsive Disorder: An Updated Systematic Review and Meta-Analysis. J. ECT.

[15] Vila-Merkle H., González-Martínez A., Campos-Jiménez R., Martínez-Ricós J., Teruel-Martí V., Blasco-Serra A., Lloret A., Celada P., Cervera-Ferri A. (2021). The Oscillatory Profile Induced by the Anxiogenic Drug FG-7142 in the Amygdala-Hippocampal Network Is Reversed by Infralimbic Deep Brain Stimulation: Relevance for Mood Disorders. Biomedicines.

[16] Borgomaneri S., Battaglia S., Garofalo S., Tortora F., Avenanti A., di Pellegrino G. (2020). State-Dependent TMS over Prefrontal Cortex Disrupts Fear-Memory Reconsolidation and Prevents the Return of Fear. Curr. Biol..

[17] Wagner T., Valero-Cabre A., Pascual-Leone A. (2007). Noninvasive Human Brain Stimulation. Annu. Rev. Biomed. Eng..

[18] Nitsche M.A., Cohen L.G., Wassermann E.M., Priori A., Lang N., Antal A., Paulus W., Hummel F., Boggio P.S., Fregni F. (2008). Transcranial Direct Current Stimulation: State of the Art 2008. Brain Stimul..

[19] Harika-Germaneau G., Heit D., Chatard A., Thirioux B., Langbour N., Jaafari N. (2020). Treating Refractory Obsessive–compulsive Disorder with Transcranial Direct Current Stimulation: An Open Label Study. Brain Behav..

[20] Bation R., Mondino M., Le Camus F., Saoud M., Brunelin J. (2019). Transcranial Direct Current Stimulation in Patients with Obsessive Compulsive Disorder: A Randomized Controlled Trial. Eur. Psychiatry.

[21] Bation R., Poulet E., Haesebaert F., Saoud M., Brunelin J. (2016). Transcranial Direct Current Stimulation in Treatment-Resistant Obsessive-Compulsive Disorder: An Open-Label Pilot Study. Prog. Neuropsychopharmacol. Biol. Psychiatry.

[22] Silva R.M.F., Brunoni A.R., Goerigk S., Batistuzzo M.C., Costa D.L.C., Diniz J.B., Padberg F., D’Urso G., Miguel E.C., Shavitt R.G. (2021). Efficacy and Safety of Transcranial Direct Current Stimulation as an Add-on Treatment for Obsessive-Compulsive Disorder: A Randomized, Sham-Controlled Trial. Neuropsychopharmacology.

[23] D’Urso G., Brunoni A.R., Mazzaferro M.P., Anastasia A., de Bartolomeis A., Mantovani A. (2016). Transcranial Direct Current Stimulation for Obsessive-Compulsive Disorder: A Randomized, Controlled, Partial Crossover Trial. Depress. Anxiety.

[24] Yoosefee S., Amanat M., Salehi M., Mousavi S.V., Behzadmanesh J., Safary V., Yoonesi A., Salehi B. (2020). The Safety and Efficacy of Transcranial Direct Current Stimulation as Add-on Therapy to Fluoxetine in Obsessive-Compulsive Disorder: A Randomized, Double-Blind, Sham-Controlled, Clinical Trial. BMC Psychiatry.

[25] Gowda S.M., Narayanaswamy J.C., Hazari N., Bose A., Chhabra H., Balachander S., Bhaskarapillai B., Shivakumar V., Venkatasubramanian G., Reddy Y.C.J. (2019). Efficacy of Pre-Supplementary Motor Area Transcranial Direct Current Stimulation for Treatment Resistant Obsessive Compulsive Disorder: A Randomized, Double Blinded, Sham Controlled Trial. Brain Stimul..

[26] Kumar S., Kumar N., Verma R. (2019). Safety and Efficacy of Adjunctive Transcranial Direct Current Stimulation in Treatment-Resistant Obsessive-Compulsive Disorder: An Open-Label Trial. Indian J. Psychiatry.

[27] Najafi K., Fakour Y., Zarrabi H., Heidarzadeh A., Khalkhali M., Yeganeh T., Farahi H., Rostamkhani M., Najafi T., Shabafroz S. (2017). Efficacy of Transcranial Direct Current Stimulation in the Treatment: Resistant Patients Who Suffer from Severe Obsessive-Compulsive Disorder. Indian J. Psychol. Med..

[28] Acevedo N., Bosanac P., Pikoos T., Rossell S., Castle D. (2021). Therapeutic Neurostimulation in Obsessive-Compulsive and Related Disorders: A Systematic Review. Brain Sci..

[29] Ouzzani M., Hammady H., Fedorowicz Z., Elmagarmid A. (2016). Rayyan-a Web and Mobile App for Systematic Reviews. Syst. Rev..

[30] Akbari S., Hassani-Abharian P., Tajeri B. (2022). The Effect of Transcranial Direct Current Stimulation (tDCS) on Cerebellum in Reduction of the Symptoms of Obsessive-Compulsive Disorder. Neurocase.

[31] Shafiezadeh S., Eshghi M., Dokhaei Z., Mohajeri H., MohammadShirazi A., Mirsadeghi S., Hasani Abharian P. (2021). Effect of Transcranial Direct Current Stimulation on Dorsolateral Prefrontal Cortex to Reduce the Symptoms of the Obsessive-Compulsive Disorder. Basic Clin. Neurosci..

[32] Ekeu-Wei I.T., Blackburn G.A., Giovannettone J. (2020). Accounting for the Effects of Climate Variability in Regional Flood Frequency Estimates in Western Nigeria. J. Water Resour. Prot..

[33] Li T., Higgins J.P.T., Deeks J.J. (2019). Collecting data. Cochrane Handbook for Systematic Reviews of Interventions.

[34] Saturnino G.B., Madsen K.H., Thielscher A. (2019). Electric Field Simulations for Transcranial Brain Stimulation Using FEM: An Efficient Implementation and Error Analysis. J. Neural Eng..

[35] Huang Y., Liu A.A., Lafon B., Friedman D., Dayan M., Wang X., Bikson M., Doyle W.K., Devinsky O., Parra L.C. (2018). Correction: Measurements and Models of Electric Fields in the In Vivo Human Brain during Transcranial Electric Stimulation. eLife.

[36] Nielsen J.D., Madsen K.H., Puonti O., Siebner H.R., Bauer C., Madsen C.G., Saturnino G.B., Thielscher A. (2018). Automatic Skull Segmentation from MR Images for Realistic Volume Conductor Models of the Head: Assessment of the State-of-the-Art. Neuroimage.

[37] Glasser M.F., Coalson T.S., Robinson E.C., Hacker C.D., Harwell J., Yacoub E., Ugurbil K., Andersson J., Beckmann C.F., Jenkinson M. (2016). A Multi-Modal Parcellation of Human Cerebral Cortex. Nature.

[38] Shephard E., Stern E.R., van den Heuvel O.A., Costa D.L.C., Batistuzzo M.C., Godoy P.B.G., Lopes A.C., Brunoni A.R., Hoexter M.Q., Shavitt R.G. (2021). Toward a Neurocircuit-Based Taxonomy to Guide Treatment of Obsessive-Compulsive Disorder. Mol. Psychiatry.

[39] Melsen W.G., Bootsma M.C.J., Rovers M.M., Bonten M.J.M. (2014). The Effects of Clinical and Statistical Heterogeneity on the Predictive Values of Results from Meta-Analyses. Clin. Microbiol. Infect..

[40] Von Hippel P.T. (2015). The Heterogeneity Statistic I(2) Can Be Biased in Small Meta-Analyses. BMC Med. Res. Methodol..

[41] Senço N.M., Huang Y., D’Urso G., Parra L.C., Bikson M., Mantovani A., Shavitt R.G., Hoexter M.Q., Miguel E.C., Brunoni A.R. (2015). Transcranial Direct Current Stimulation in Obsessive-Compulsive Disorder: Emerging Clinical Evidence and Considerations for Optimal Montage of Electrodes. Expert Rev. Med. Devices.

[42] Da Silva R.d.M.F., Batistuzzo M.C., Shavitt R.G., Miguel E.C., Stern E., Mezger E., Padberg F., D’Urso G., Brunoni A.R. (2019). Transcranial Direct Current Stimulation in Obsessive-Compulsive Disorder: An Update in Electric Field Modeling and Investigations for Optimal Electrode Montage. Expert Rev. Neurother..

[43] Brunoni A.R., Sampaio-Junior B., Moffa A.H., Aparício L.V., Gordon P., Klein I., Rios R.M., Razza L.B., Loo C., Padberg F. (2019). Noninvasive Brain Stimulation in Psychiatric Disorders: A Primer. Braz. J. Psychiatry.

[44] Carmi L., Tendler A., Bystritsky A., Hollander E., Blumberger D.M., Daskalakis J., Ward H., Lapidus K., Goodman W., Casuto L. (2019). Efficacy and Safety of Deep Transcranial Magnetic Stimulation for Obsessive-Compulsive Disorder: A Prospective Multicenter Randomized Double-Blind Placebo-Controlled Trial. Am. J. Psychiatry.

[45] Roth Y., Zangen A. (2014). Reaching Deep Brain Structures: The H-coils. Transcranial Magnetic Stimulation.

[46] Hyde J., Carr H., Kelley N., Seneviratne R., Reed C., Parlatini V., Garner M., Solmi M., Rosson S., Cortese S. (2022). Efficacy of Neurostimulation across Mental Disorders: Systematic Review and Meta-Analysis of 208 Randomized Controlled Trials. Mol. Psychiatry.

[47] Brunoni A.R., Ekhtiari H., Antal A., Auvichayapat P., Baeken C., Bensenor I.M., Bikson M., Boggio P., Borroni B., Brighina F. (2022). Digitalized Transcranial Electrical Stimulation: A Consensus Statement. Clinic Neurophysiol..

[48] Volpato C., Piccione F., Cavinato M., Duzzi D., Schiff S., Foscolo L., Venneri A. (2013). Modulation of Affective Symptoms and Resting State Activity by Brain Stimulation in a Treatment-Resistant Case of Obsessive-Compulsive Disorder. Neurocase.

[49] Brunoni A.R., Amadera J., Berbel B., Volz M.S., Rizzerio B.G., Fregni F. (2011). A Systematic Review on Reporting and Assessment of Adverse Effects Associated with Transcranial Direct Current Stimulation. Int. J. Neuropsychopharmacol..

[50] Razza L.B., De Smet S., Moffa A., Sudbrack-Oliveira P., Vanderhasselt M.-A., Brunoni A.R. (2021). Follow-up Effects of Transcranial Direct Current Stimulation (tDCS) for the Major Depressive Episode: A Systematic Review and Meta-Analysis. Psychiatry Res..

[51] Riley R.D., Lambert P.C., Abo-Zaid G. (2010). Meta-Analysis of Individual Participant Data: Rationale, Conduct, and Reporting. BMJ.

[52] Guzick A.G., Schweissing E., Tendler A., Sheth S.A., Goodman W.K., Storch E.A. (2022). Do Exposure Therapy Processes Impact the Efficacy of Deep TMS for Obsessive-Compulsive Disorder?. J. Obs. Compuls. Relat. Disord..

