# Supplementary material for: The Effects of Transcranial Direct Current Stimulation in Obsessive–Compulsive Disorder Symptoms: A Meta-Analysis and Integrated Electric Fields Modeling Analysis
Source: Biomedicines. 2022 Dec 29;11(1):80. doi: 10.3390/biomedicines11010080 (PMC9855366; doi:10.3390/biomedicines11010080)
Supplement: Supplementary file 1 [file biomedicines-11-00080-s001.zip › biomedicines-1919351-supplementary.pdf]

SUPPLEMENTARY MATERIAL

The Effects of Transcranial Direct Current Stimulation in Obsessive–Compulsive Disorder Symptoms: A Meta-Analysis and Integrated Electric Fields Modeling Analysis

Bianca Silva Pinto [1], Beatriz Araújo Cavendish [1], Pedro Henrique Rodrigues da Silva [1], Paulo Jeng Chian Suen [1], Kalian Almeida Pereira Marinho [1,2], Leandro da Costa Lane Valiengo [1,2,3], Marie-Anne Vanderhasselt [4,5], André Russowsky Brunoni [1,3,6] and Laís Boralli Razza [1,4,5,*]

[1] Serviço Interdisciplinar de Neuromodulação (SIN), Instituto de Psiquiatria do Hospital das Clínicas HCFMUSP, Faculdade de Medicina, Universidade de São Paulo, São Paulo 05403-903, Brazil
[2] Programa de Fisiopatologia Experimental, Faculdade de Medicina, Universidade de São Paulo, São Paulo 05403-903, Brazil
[3] Laboratório de Neurociências (LIM-27), Instituto de Psiquiatria do Hospital das Clínicas HCFMUSP, Faculdade de Medicina, Universidade de São Paulo, São Paulo 05403-903, Brazil
[4] Department of Head and Skin, Psychiatry and Medical Psychology, Ghent University Hospital, Corneel Heymanslaan, 9000 Ghent, Belgium
[5] Ghent Experimental Psychiatry (GHEP) Lab, 9000 Ghent, Belgium
[6] Departamento de Clínica Médica, Faculdade de Medicina, Universidade de São Paulo, São Paulo 05403-903, Brazil
* Correspondence: lais.razza@ugent.be

supplementary file S1 Syntax used for literature search

supplementary file S2 - Risk of bias from RoB2 randomized trials

supplementary file S3 - Risk of bias In non-randomised studies - of Interventions(ROBINS-I) tool

supplementary file S4 - Funnel Plot for the assessment of publication bias in this meta-analysis

supplementary file S5 - Forest plot of active and sham groups

supplementary file S6 - Electric field Modeling

supplementary file S7 - Graphical representation of the linear regressions performed for the electric field modeling analysis.

supplementary file S1

Syntax used for literature search

PubMed/MEDLINE

The full search string reads:
(((“OCD” OR “'obsessive compulsive disorder”)) AND (“transcranial direct current stimulation” OR “tDCS”)) AND (“clinical trial”)

EMBASE

The full search string reads:
((OCD:ti,ab,kw OR obsessive) AND compulsive AND disorder:ti,ab,kw) AND (((Tdcs:ti,ab,kw OR transcranial) AND direct AND current AND stimulation:ti,ab,kw OR electric) AND stimulation:ti,ab,kw) AND (clinical AND trial':ti,ab,kw)

Cochrane Library

The full search string reads:
“OCD” OR “'obsessive compulsive disorder” in Title Abstract Keyword AND “transcranial direct current stimulation” OR “tDCS” in Title Abstract Keyword AND “clinical trial” in Title Abstract Keyword- (Word variations have been searched)

Web of Science

The full search string reads:
All Fields: ('tDCS” OR “transcranial direct current stimulation” OR “electricstimulation”) AND All Fields: (“OCD” OR “obsessive compulsive disorder”) AND All Fields: (“clinical trial”)

supplementary file S2

Table S1. Risk of bias - randomized trials

Bias domain RoB2	Bation et al. 2019	Gowda et al. 2019	Da Silva et al. 2020	Yoosefee et al. 2020	Shafiezadeh et. al 2021	Akbari et al 2022
Pre-intervention domains						
Randomization process	Low	Low	Low	Low	Some concers[2]	Some concers[2]
At-intervention domain						
Deviations from intended interventions	Low	Some concers[1]	Low	Low	Low	High[3]
Post-intervention domains						
Missing outcome data	Low	Low	Low	Low	Low	High[3]
Measurement of the outcome	Low	Low	Low	Low	High[4]	High[4]
Selection of the reported result	Low	Low	Low	Low	Some concers[5]	Some concers[5]
Overall Bias	Low	Some concers[1]	Low	Low	High[4]	High[3]

Note: Studies (Shafiezadeh et al. 2021; Akbari et al. 2022) were excluded from our analysis because they had a high risk of bias and lacked methodological issues(1,2) .

1. There is no specification whether the final analysis include the data of the one participant who dropped-out
2. The manuscript does not report how the randomization was performed.
3.There is a deviation between the informed electrode positions and the target specification. For instance, the authors inform that electrodes were positioned over the supplementary motor area and over the right cerebellum. However, the targets informed are C3/C4 - which corresponds to the primary motor cortex - and O2, which corresponds to the right orbital area. Authors were contacted for further clarification, but no reply was obtained.
4. Outcome measurement data is lacking at the endpoint.
5. No specification about the blinding was given - whether the evaluator was aware or not of the condition delivered.

supplementary file S3

Table S2. Risk Of Bias - Non-randomised Studies

**Bias domain ROBINS-I**	**Bation et al. 2016**	**Najadi et al 2017**	**Germaneau et al. 2020**	**Kumar et al. 2021**	**D´Urso et al. 2016**
Pre-intervention domains					
Confounding	Low[1]	Low	Low[1]	Low[1]	Low[1]
Selection of participants into the study	Low	Low	Low	Low	Low
At-intervention domain					
Classification of interventions	Low	High[2]	Low	Low	Low
Post-intervention domains					
Deviations from intended interventions	Low	Low	Low	Low	Low
Missing data	Low	Low	Low	Low	Low
Measurement of the outcome	Low	Some Concers[2]	Low	Low	Low
Selection of the reported result	Low	Some Concers[2]	Low	Low	Low
Overall Bias	Moderate[1]	High[2]	Moderate[1]	Moderate[1]	Moderate[1]

1. Evaluators were aware of the intervention that participants received
2. Discrepant information regarding the intensity of the current used for the tDCS session in the acute phase.

supplementary file S4

Electric field Modeling

For electric field modeling for the studies by Gowda et al. 2019 and Germeneau et al. 2020, the electrodes were positioned horizontally and vertically in order to observe whether there would be stronger electric fields in the regions of interest. In the study by Germaneau et al. 2020, a difference was observed when the electrodes were placed horizontally, as the electric fields were stronger in the specific ROIs when the electrodes were placed horizontally. However, in the study by Gowda et al. 2019 no differences were found.

Table S3. Differences observed in the electric fields of Gowda et al. 2019 and Germeneau et al. 2020 (3,4)

ROI	Germaneau et al. 2020 Mean (p)	Gowda et al. 2019 Mean (p)
**Left DLPFC**	0.17(<0.001)	0.17 (<0.001)
**Left Insula**	0.11(<0.001)	0.12 (<0.001)
**Left SMA**	0.11(<0.001)	-
**Right SMA**	0.12 (<0.001)	-
**Left pre-SMA**	0.14 (<0.001)	0.15 (<0.001)
**Right pre-SMA**	0.19 (< 0.001)	0.15 (<0.001)
**Right Insula**	0.14 (<0.009)	-
**RighT ACC**	0.16 (<0.051)	-

Gowda et al. 2019.First figure shows electrodes pointing towards Cz and the second figure shows the electrodes placed horizontally (3).

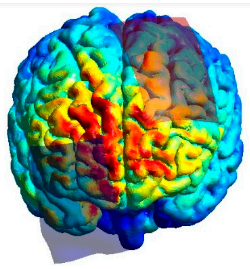

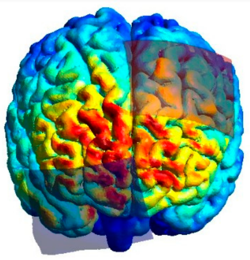

Germeneau et al. 2020. First figure shows electrodes pointing towards Cz and the second figure shows the electrodes placed horizontally (4).

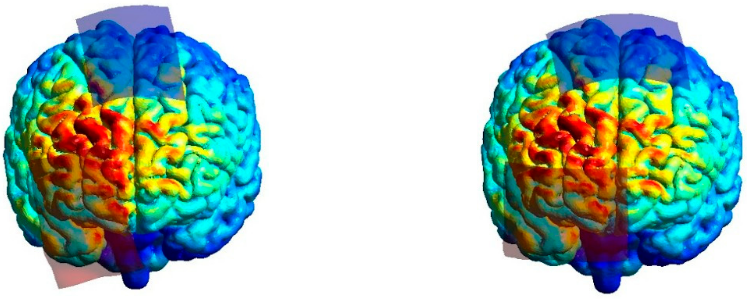

Table S4. Each region of interest was composed of several sub-regions included in the Glasser et al. 2016 atlas (5).

DLPFC	Insula	ACC	SMA	Pre-SMA
lh.8C	lh.AVI	lh.p24	lh.SCEF	lh.6r
rh.8C	rh.AVI	rh.p24	rh.SCEF	rh.6r
lh.8Av	lh.MI	lh.a24pr	lh.6ma	lh.6v
rh.8Av	rh. MI	rh.a24pr	rh.6ma	rh.6v
lh.i6-8	lh.FOP3	lh.p24pr	lh.6mp	—
rh.i6-8	rh.FOP3	rh.p24pr	rh.6mp	—
lh.s6-8	lh.AAIC	—	—	—
rh.s6-8	rh.AAIC	—	—	—
lh.SFL	lh.PoI1	—	—	—
rh.SFL	rh.PoI1	—	—	—
lh.9p	lh.PoI2	—	—	—
rh.9p	rh.PoI2	—	—	—
lh.9a	lh.Ig	—	—	—
rh.9a	rh.Ig	—	—	—
lh.8Ad	lh.FOP2	—	—	—
rh.8Ad	rh.FOP2	—	—	—
lh.p9-46v	—	—	—	—
rh.p9-46v	—	—	—	—
lh.a9-46v	—	—	—	—
rh.a9-46v	—	—	—	—
lh.46	—	—	—	—
rh.46	—	—	—	—
lh.9-46d	—	—	—	—
rh.9-46d	—	—	—	—

Abbreviation: Anterior Cingulate CortexACC: Anterior Cingulate Cortex, DLPFC: Dorsolateral Prefrontal Cortex, SMA: Supplementary Motor Area

supplementary file S5

Figure S1. Funnel Plot for the assessment of publication bias in this meta- analysis

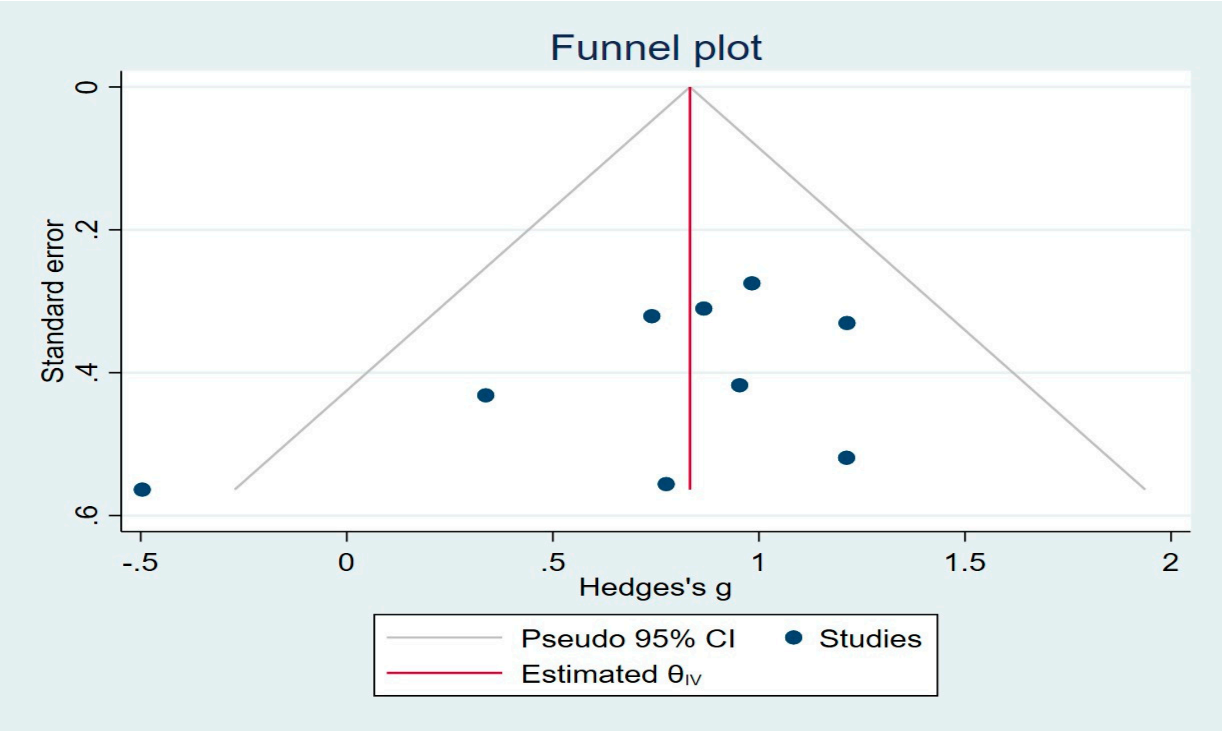

supplementary file S6

Forest plot of active and sham groups

Comparisons between baseline and endpoint of both active and sham groups showed that both presented improvements at the endpoint, with a larger effect size for the active group.

Figure S2A. Forest plot of the active RCT groups comparing the baseline and theendpoint Y-BOCS scores.

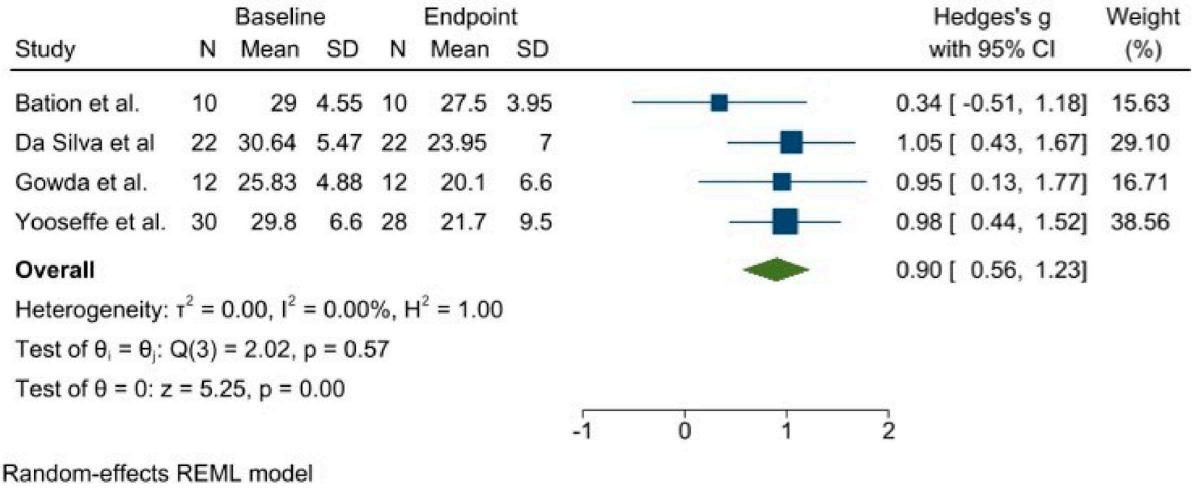

Figure S2B. Forest plot of the sham RCT groups comparing the baseline and theendpoint Y-BOCS scores.

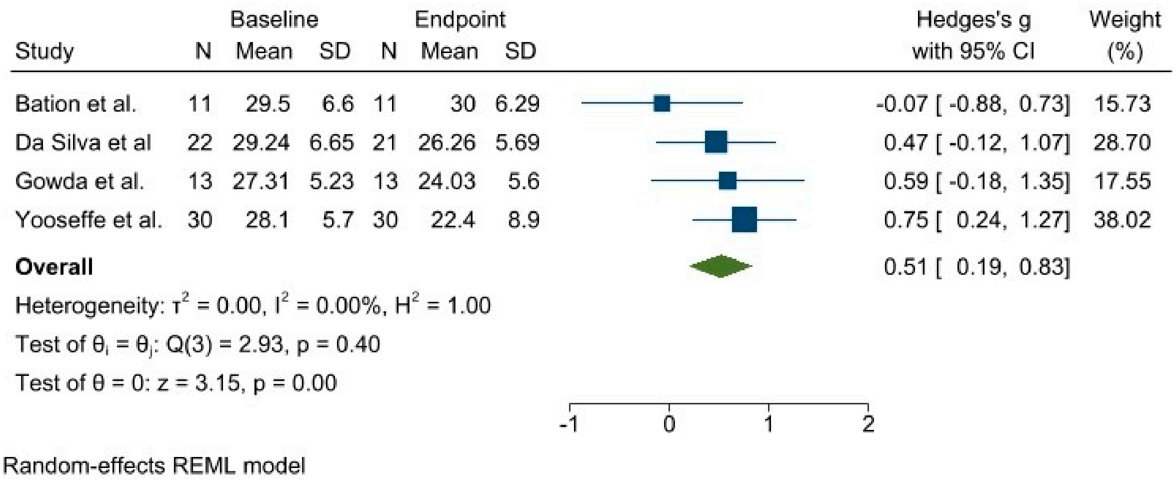

supplementary file S7

Graphical representation of the linear regressions performed for the electric field modeling analysis.

Left DLPFC

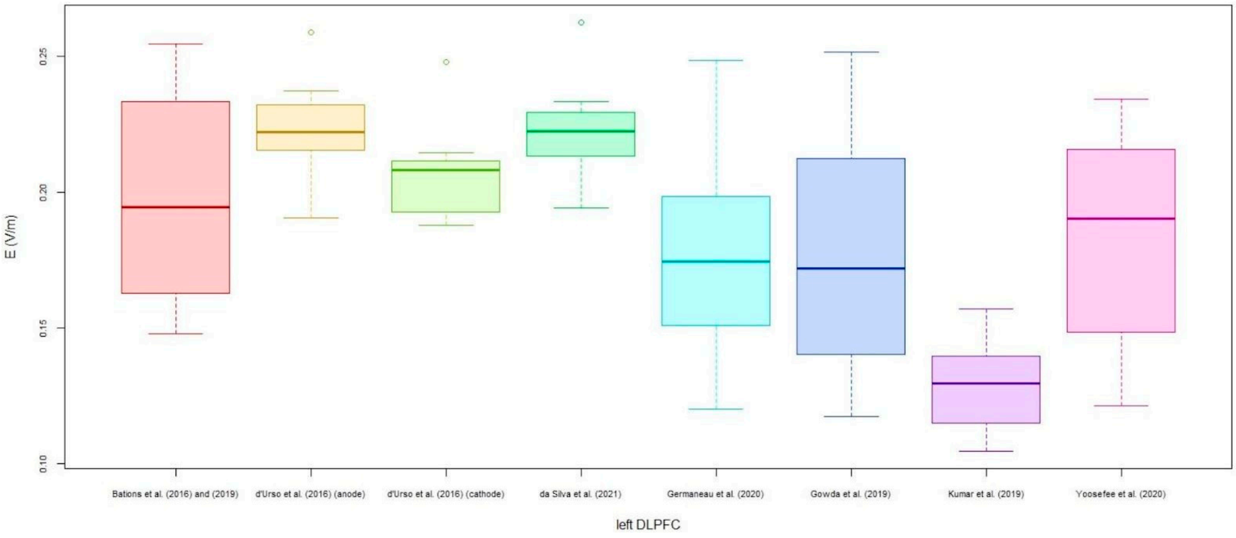

Abbreviation: Dorsolateral Prefrontal Cortex (DLPFC)

Right DLPFC

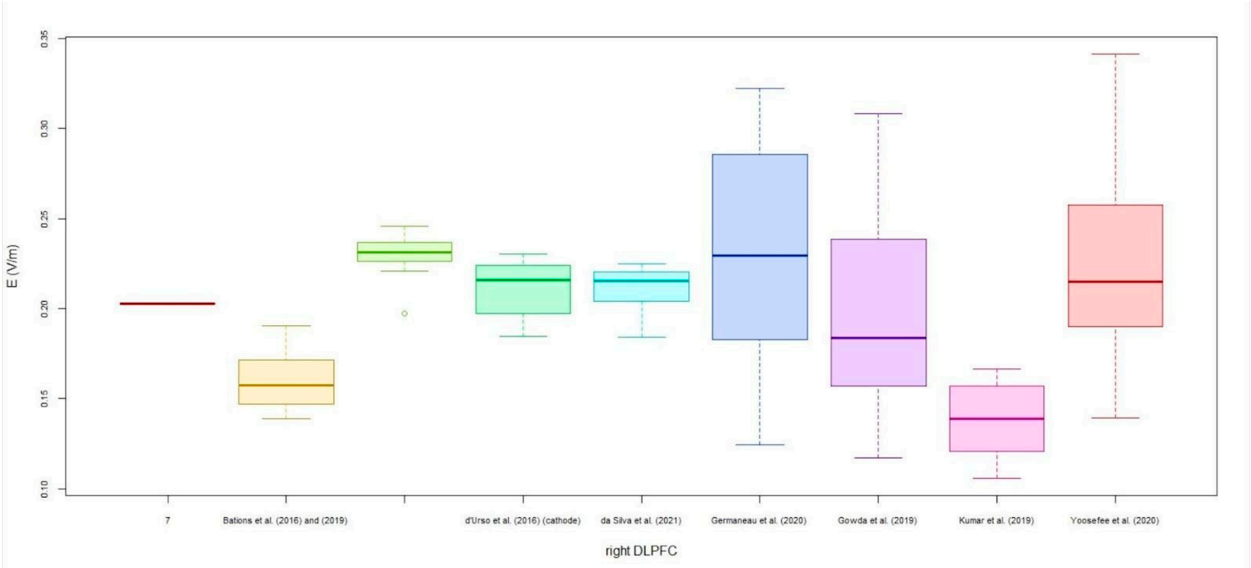

Abbreviation: Dorsolateral Prefrontal Cortex (DLPFC

Left Insula

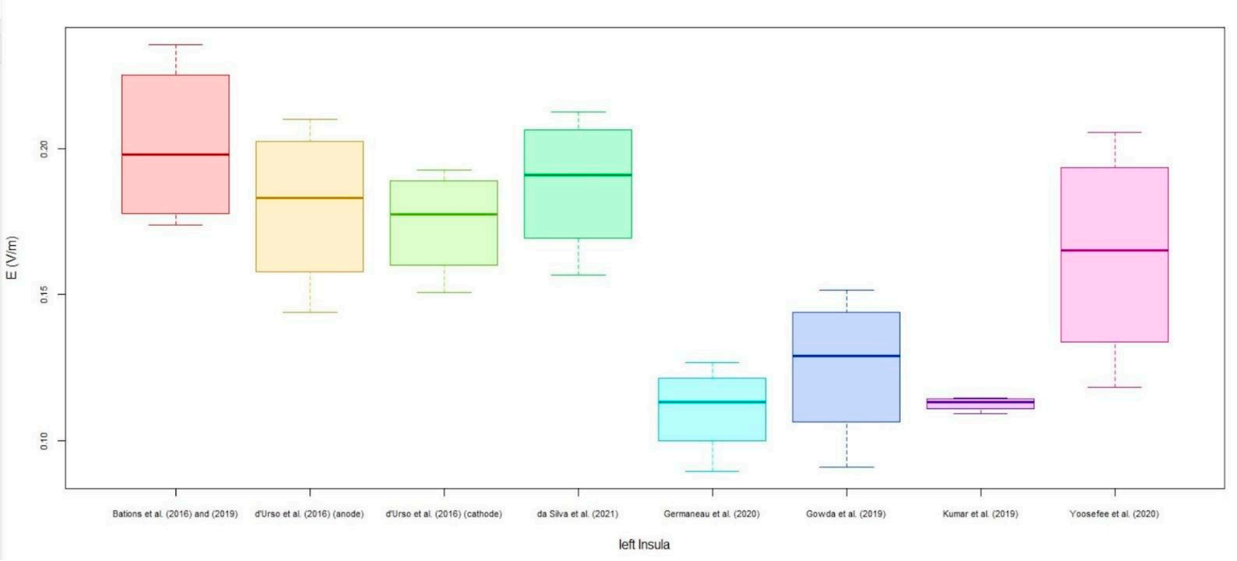

Right Insula

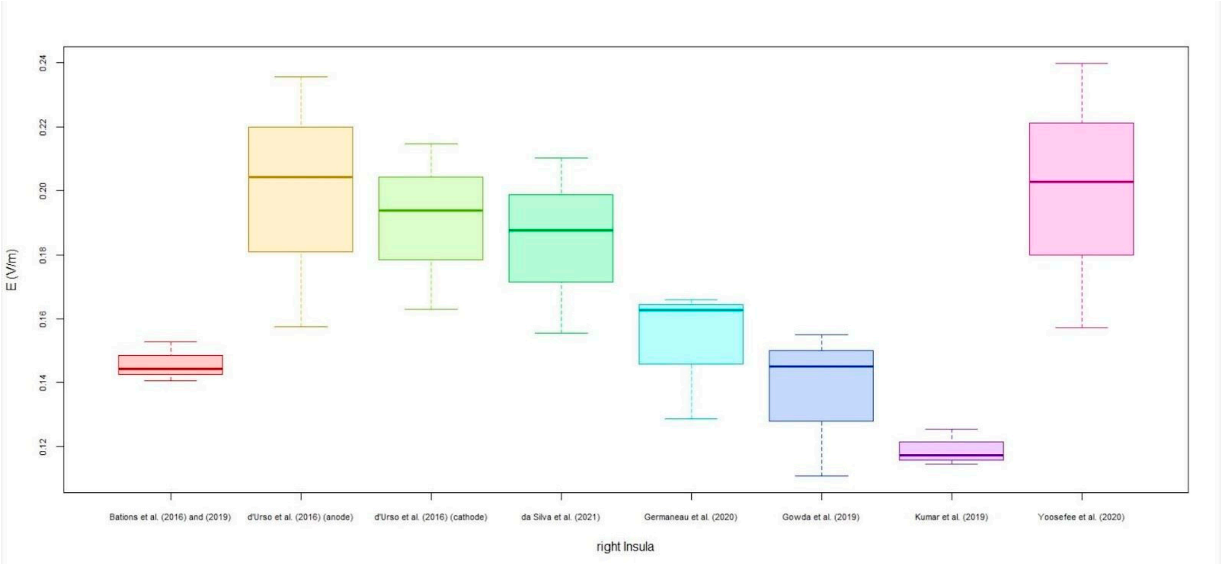

Left ACC

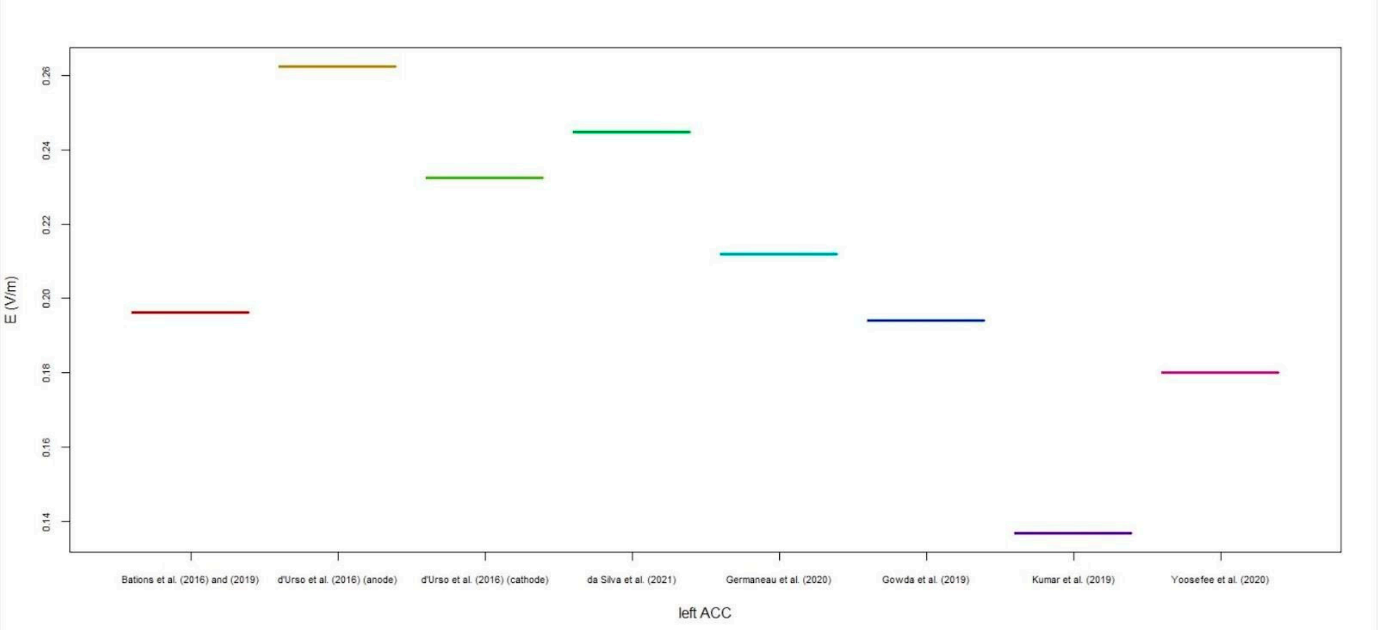

Abbreviation: Acc: Anterior Cingulate Cortex

Right ACC

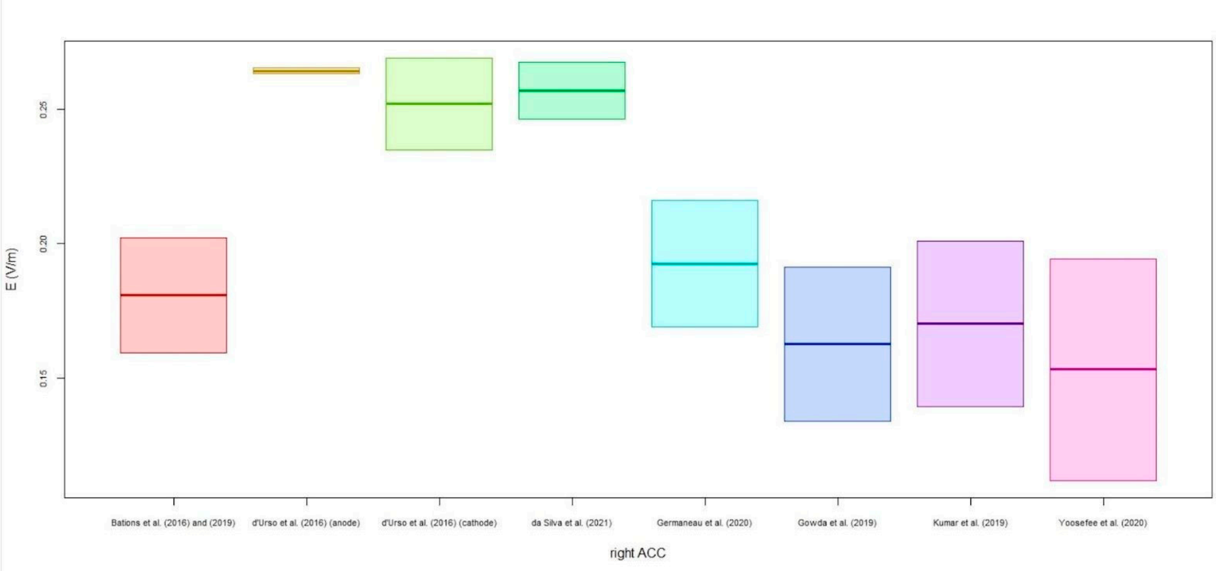

Abbreviation: Acc: Anterior Cingulate Cortex

Left SMA

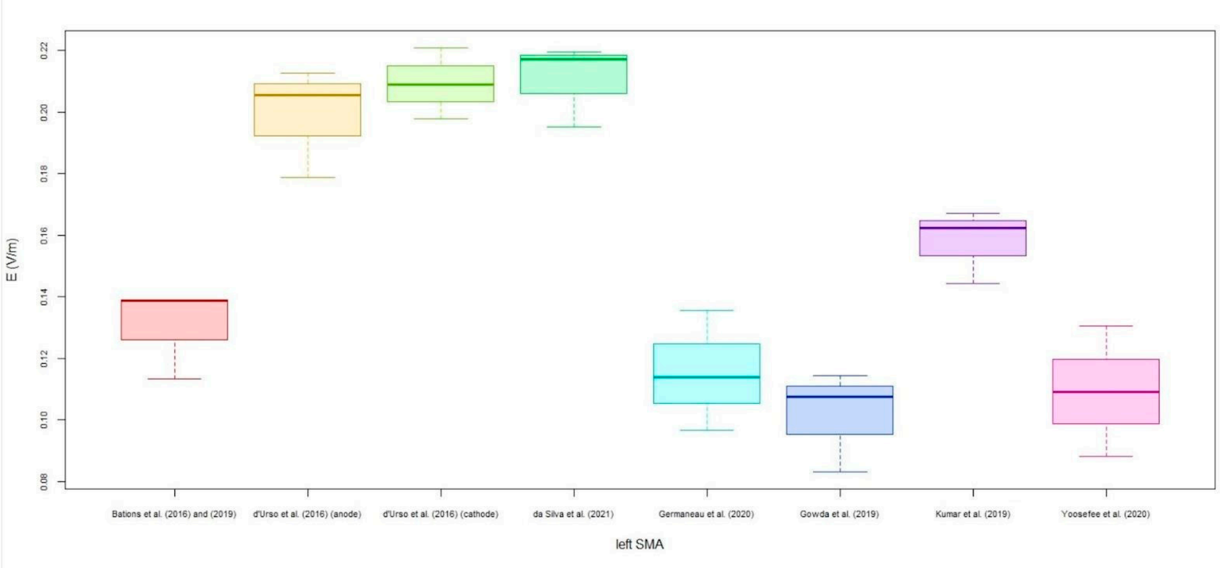

Abbreviation: SMA: Supplementary motor area

Right SMA

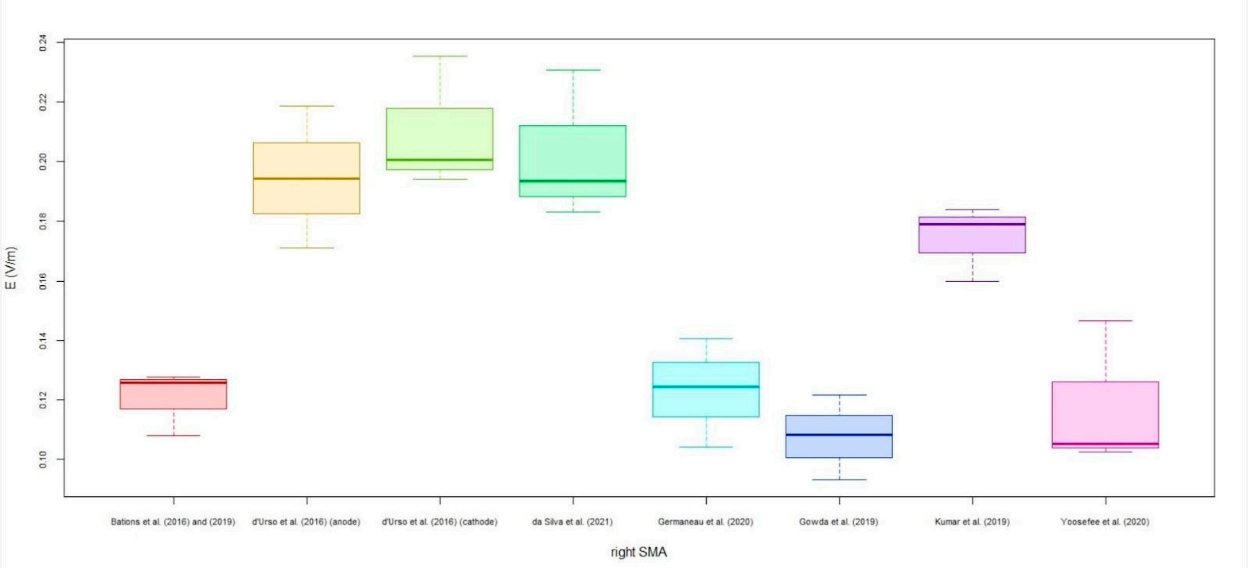

Abbreviation: SMA: Supplementary motor area

Left Pre SMA

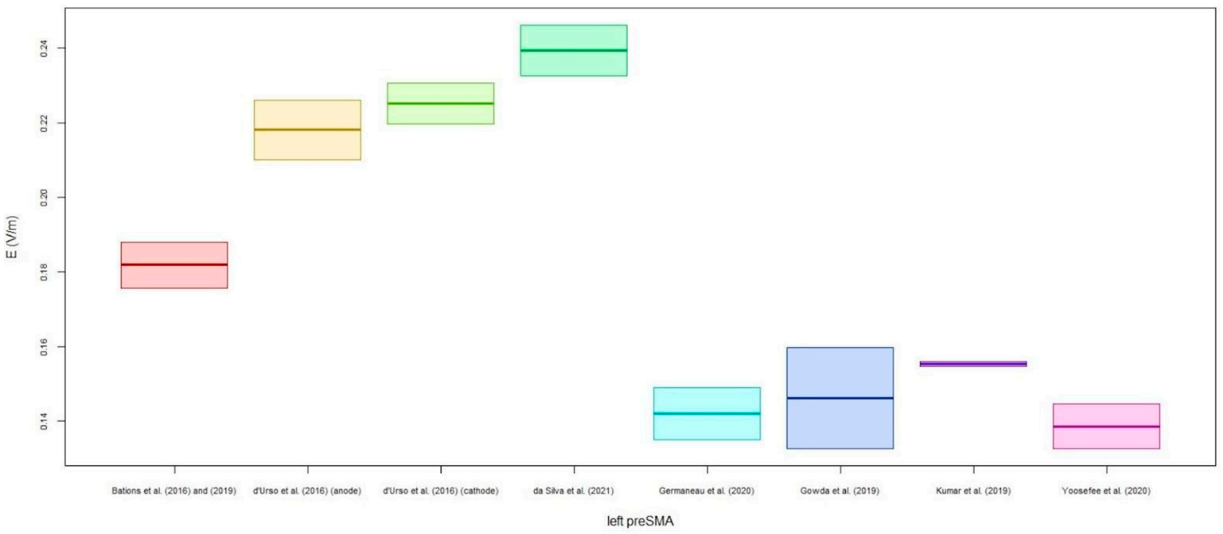

Abbreviation: Supplementary Motor Area (SMA)

Right Pre SMA

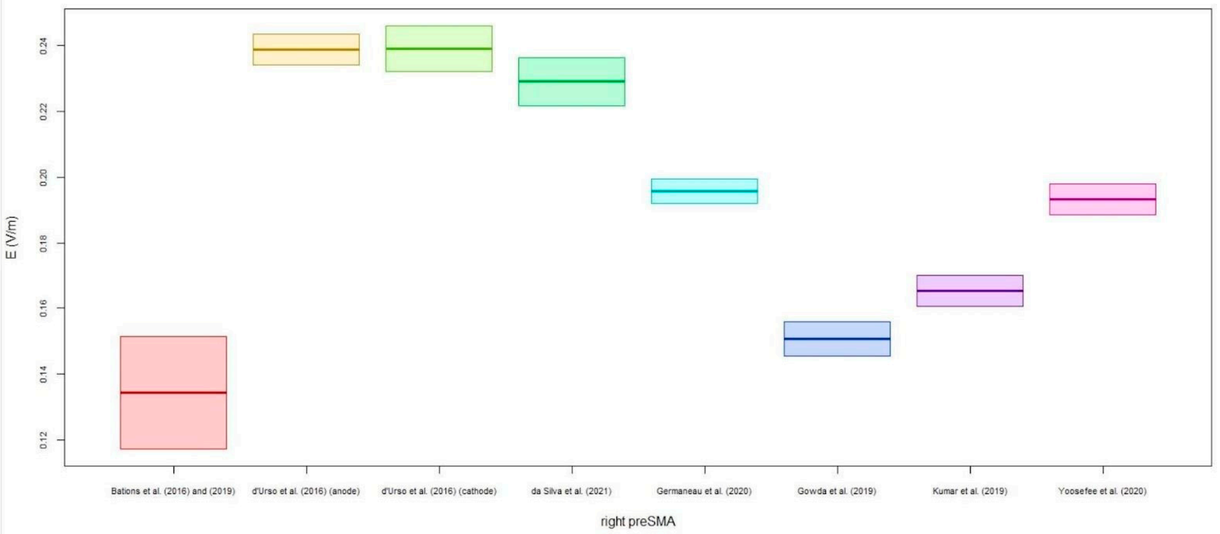

Abbreviation: SMA: Supplementary motor area

References

1. Akbari S, Hassani-Abharian P, Tajeri B. The effect of transcranial direct current stimulation (tDCS) on cerebellum in reduction of the symptoms of obsessive-compulsive disorder. Neurocase. 2022 Apr;28(2):135–9.

2. Shafiezadeh S, Eshghi M, Dokhaei Z, Mohajeri H, MohammadShirazi A, Mirsadeghi S, et al. Effect of Transcranial Direct Current Stimulation on Dorsolateral Prefrontal Cortex to Reduce the Symptoms of the Obsessive-Compulsive Disorder. Basic Clin Neurosci. 2021 Sep;12(5):675–80.

3. Gowda SM, Narayanaswamy JC, Hazari N, Bose A, Chhabra H, Balachander S, et al. Efficacy of pre-supplementary motor area transcranial direct current stimulation for treatment resistant obsessive compulsive disorder: A randomized, double blinded, sham controlled trial. Brain Stimul. 2019 Jul;12(4):922–9.

4. Harika-Germaneau G, Heit D, Chatard A, Thirioux B, Langbour N, Jaafari N. Treating refractory obsessive-compulsive disorder with transcranial direct current stimulation: An open label study. Brain Behav. 2020 Jul;10(7):e01648.

5. Glasser MF, Coalson TS, Robinson EC, Hacker CD, Harwell J, Yacoub E, et al. A multi-modal parcellation of human cerebral cortex. Nature. 2016 Aug 11;536(7615):171–8.
